# Alcohol consumption patterns and risk of HIV transmission among men who have sex with men living with HIV in Chongqing, southwestern China

**DOI:** 10.3389/fpubh.2025.1629368

**Published:** 2025-10-15

**Authors:** Huailiang Chen, Hui Fan, Panhao Yang, Yingxin Luo, Jin Chen

**Affiliations:** ^1^Department of Infection Control, Sichuan Tianfu New Area People's Hospital, Chengdu, Sichuan, China; ^2^Department of Epidemiology and Health Statistics, School of Public Health, North Sichuan Medical College, Nanchong, Sichuan, China; ^3^School of Basic Medical Sciences, Southwest Medical University, Luzhou, Sichuan, China; ^4^School of Public Health, Chengdu Medical College, Chengdu, Sichuan, China; ^5^Department of Medical Records Management, The People’s Hospital of Tongliang District, Chongqing, China

**Keywords:** alcohol consumption patterns, antiretroviral treatment adherence, sexual risk-taking behaviors, men who have sex with men, human immunodeficiency virus

## Abstract

**Introduction:**

There has been limited research on alcohol consumption patterns and their specific risks for the transmission of human immunodeficiency virus (HIV) and sexually transmitted infections (STIs) among men who have sex with men (MSM) living with HIV. The aim of this study was to examine the effects of alcohol consumption patterns on antiretroviral treatment (ART) adherence and sexual risk-taking behaviors among MSM living with HIV in Chongqing, a municipality with the highest HIV prevalence among MSM in China.

**Methods:**

An institutionally based cross-sectional survey was conducted between 2022 and 2023 in Chongqing, China. The Alcohol Use Disorders Identification Test-Consumption (AUDIT-C) was administered to evaluate alcohol consumption patterns. Multivariate logistic regression analysis was employed to examine the associations between alcohol consumption patterns, ART adherence, and sexual risk-taking behaviors.

**Results:**

Of the 1,501 MSM living with HIV, the prevalence of alcohol use, hazardous drinking, and binge drinking was 60.2, 24.9 and 43.6%, respectively. Multivariate logistic regression analysis revealed that alcohol use, hazardous drinking, and binge drinking were significantly associated with a higher likelihood of ART non-adherence (adjusted odds ratio [aOR] = 1.89, 95% confidence interval [CI]: 1.08–3.30; aOR = 3.43, 95% CI: 2.11–5.58; aOR = 2.17, 95% CI: 1.30–3.62), having multiple sex partners (aOR = 1.62, 95% CI: 1.27–2.06; aOR = 1.49, 95% CI: 1.11–1.99; aOR = 1.65, 95% CI: 1.29–2.11), engaging in commercial sex (aOR = 2.50, 95% CI: 1.56–3.99; aOR = 3.21, 95% CI: 2.16–4.75; aOR = 3.11, 95% CI: 2.03–4.75), and participating in group sex (aOR = 1.73, 95% CI: 1.05–2.83; aOR = 1.79, 95% CI: 1.14–2.80; aOR = 1.77, 95% CI: 1.12–2.80). Alcohol use and binge drinking were associated with higher odds of casual sex (aOR = 1.31, 95% CI: 1.03–1.66; aOR = 1.52, 95% CI: 1.18–1.95). Hazardous drinking and binge drinking were associated with condomless internal ejaculation (CIE) (aOR = 1.37, 95% CI: 1.03–1.83; aOR = 1.44, 95% CI: 1.11–1.86).

**Conclusion:**

High levels of alcohol use, hazardous drinking, and binge drinking are observed among MSM living with HIV in Chongqing. Given the significant associations between alcohol consumption, ART non-adherence, and sexual risk-taking behaviors, public health strategies in Chongqing and similar settings may benefit from screening and interventions to address alcohol consumption among MSM living with HIV.

## 1 Introduction

Alcohol consumption continues to be one of the major public health concerns globally, contributing significantly to both mortality and morbidity. According to the World Health Organization (WHO), alcohol is responsible for 4.6% of the overall disease burden and 6.7% of all premature deaths (1). Beyond its direct health consequences, alcohol consumption is often linked to risky behaviors, which can further worsen the burden of infectious diseases (1–3). Among these, unprotected sex is a key risk factor for the transmission of human immunodeficiency virus (HIV) and other sexually transmitted infections (STIs), particularly among vulnerable populations such as men who have sex with men (MSM) (4–7).

MSM are recognized as a high-risk group for both HIV acquisition and transmission. In the United States, MSM account for more than two-thirds of new HIV diagnoses (8). In the European Union/European Economic Area (EU/EEA), male-to-male sexual contact represents the largest proportion of new HIV diagnoses (46.7%) (9). A similar trend is observed in China, where over a quarter of new HIV cases are attributed to male homosexual transmission (10). Over the past decade, MSM have emerged as the second-largest group among new HIV diagnoses in China, with regions such as Chongqing and Sichuan experiencing some of the highest HIV prevalence (11).

Alcohol consumption is common among MSM in these regions, and studies suggest it is linked to higher odds of sexual risk-taking behaviors, including inconsistent condom use, multiple sexual partners, and involvement in commercial sex (12–14). These behaviors significantly elevate the risk of HIV exposure and transmission. Moreover, alcohol consumption among MSM living with HIV presents additional challenges that hinder adherence to antiretroviral treatment (ART). Since ART adherence is crucial for achieving viral suppression, preventing disease progression, and reducing HIV transmission risk, alcohol consumption among MSM living with HIV may present significant challenges to both health management and HIV prevention within this group (15–17).

Although these associations between alcohol consumption and risky behaviors are well-documented, much of the research on alcohol use among MSM has been conducted in Western countries. Furthermore, existing literature typically examines the effects of alcohol consumption on either sexual risk-taking behaviors or ART adherence separately. Few studies have explored the combined impact of alcohol consumption on both ART adherence and sexual risk-taking behaviors. In China, while several studies have explored the relationship between alcohol consumption and sexual risk-taking behaviors among general MSM (2, 6, 12, 14), there remains a significant gap in understanding how specific alcohol consumption patterns (such as hazardous drinking or binge drinking) impact ART adherence and sexual risk-taking behaviors in MSM living with HIV.

This study seeks to address these gaps by exploring the relationship between alcohol consumption patterns, ART adherence, and sexual risk-taking behaviors among MSM living with HIV in Chongqing, China. By focusing on this under-researched population, the study will provide important insights into how alcohol consumption influences ART adherence and sexual risk-taking behaviors. Ultimately, the findings may inform the development of targeted interventions to reduce alcohol consumption and improve health outcomes for MSM in Chongqing and similar settings.

## 2 Methods

### 2.1 Participants

An institutionally based cross-sectional study was conducted in Chongqing, China, from June 2022 to June 2023. Participants were recruited through a convenience sampling approach in collaboration with two community-based organizations (CBOs), which operate under the guidance of local Centers for Disease Control and Prevention (CDC). Both CBOs are committed to delivering healthcare and counseling services to individuals living with HIV and offer free testing and counseling services for high-risk populations. Each CBO is managed by a team of 2 to 3 MSM, who are actively involved in outreach activities within the community.

Eligible participants were required to meet the following criteria: (1) biologically male; (2) aged 18 years or older; (3) having engaged in male-to-male sexual contact in the past 12 months; and (4) having a confirmed diagnosis of HIV. CBO staff screened individuals receiving HIV care and counseling services to identify those who met the inclusion criteria. Those eligible were invited to participate in the study. Prior to data collection, all potential participants were briefed on the study’s objectives, procedures, and confidentiality protocols. MSM who provided informed consent were then asked to complete a self-administered questionnaire.

### 2.2 Data collection

This study employed the Wenjuanxing platform, a widely used online survey tool in China, for data collection. First, the researchers registered an account on the Wenjuanxing website and uploaded the electronic version of the questionnaire. A Quick Response (QR) code linking to the online survey was then generated and distributed to eligible participants by staff members of CBOs. Participants were instructed to scan the QR code using the WeChat app. Upon scanning, they were directed to a landing page displaying the informed consent form. The consent form emphasized confidentiality, explained that participation would not affect access to services, and clarified that data would be used only for research purposes, to encourage candid reporting. Participants were required to read the consent form and indicate their agreement by selecting the “Agree” option before proceeding to complete the self-administered questionnaire. During survey design, all questions were set as required, and respondents were informed that they had to answer each question to submit. The survey was designed to take approximately 8 to 12 min.

The survey collected detailed information on participants’ sociodemographic characteristics, which included age (grouped as 18–25 years, 26–35 years, or ≥36 years), marital status (married/cohabiting with a female partner or unmarried/divorced/widowed), household registration status (classified as “migrant” for those registered in municipalities or provinces outside Chongqing, and “non-migrant” for those with household registration within Chongqing municipality), educational attainment (categorized as “senior high school or lower” or “college or higher”), employment status (full-time or part-time/other), and individual monthly income [≤5,000 or >5,000 Chinese Yuan (CNY)].

Participants were also required to provide information regarding their HIV status. The duration since HIV diagnosis was grouped into ≤1 year, 2–4 years, or ≥5 years. HIV viral load in the past year was categorized into three groups: <1,000 copies/mL, ≥1,000 copies/mL and “not tested.” Additionally, MSM participants were asked to report their recreational drug use in the previous 6 months. The substances included in the survey were poppers, crystal methamphetamine, 5-MeO-DIPT, 3,4-methylenedioxymethamphetamine (MDMA), “magu” (a tablet containing methamphetamine and caffeine), ketamine, gamma-hydroxybutyrate (GHB), cannabis, heroin, and cocaine.

#### 2.2.1 Assessment of psychological symptoms and perceived stigma

Several validated instruments were used to assess psychological symptoms and perceived stigma. Depressive symptoms were evaluated using the Patient Health Questionnaire-9 (PHQ-9), a 9-item scale designed to evaluate the frequency of depressive symptoms within the previous 2 weeks. Total scores on the PHQ-9 range from 0 to 27, with a score of 5 or higher indicating the presence of depressive symptoms (18). Anxiety symptoms were screened using the Generalized Anxiety Disorder-7 (GAD-7), a 7-item scale that evaluates anxiety symptoms over the previous 2 weeks. Total scores on the GAD-7 range from 0 to 21, with a score greater than 4 considered indicative of clinically significant anxiety symptoms (19). In this study, both the PHQ-9 (Cronbach’s alpha = 0.851) and GAD-7 (Cronbach’s alpha = 0.861) demonstrated good internal consistency.

Perceived HIV-related stigma was assessed using the 12-item short version of the HIV stigma scale, which measures the stigma and discrimination experienced by individuals living with HIV. Total scores on the scale range from 12 to 48, with higher scores reflecting greater perceived stigma (20). The Cronbach’s alpha for this scale in the current study was 0.892, indicating excellent internal reliability.

#### 2.2.2 Alcohol consumption patterns

Alcohol consumption patterns, including alcohol use, hazardous drinking, and binge drinking, were assessed using the Alcohol Use Disorders Identification Test-Consumption (AUDIT-C) (21). The AUDIT-C is a validated screening tool derived from the full Alcohol Use Disorders Identification Test (AUDIT), widely used to identify alcohol use disorders across diverse populations and cultural settings (22). The AUDIT-C focuses on the first three questions of the AUDIT, which assess the frequency of alcohol consumption, the typical quantity consumed, and the frequency of binge drinking. Each item in the AUDIT-C is scored from 0 to 4, resulting in a total score range of 0 to 12. Previous studies have validated the AUDIT-C as a reliable and effective instrument for identifying alcohol misuse among MSM (2, 6).

In this study, alcohol consumption was defined as the intake of any alcoholic beverage, including liquor, beer, wine, and other alcoholic drinks. A “standard drink” was defined as any beverage containing approximately 10 grams of pure alcohol (22). Hazardous drinking was defined as an AUDIT-C total score of 4 or higher, consistent with established thresholds used in previous research (6). Binge drinking was defined as consuming 6 or more standard drinks (i.e., 60 grams of pure alcohol) in a single drinking episode, aligning with WHO guidelines for binge drinking (22). To facilitate accurate estimation of alcohol intake, participants were provided with examples of commonly consumed alcoholic beverages in the study area along with their corresponding alcohol content. For instance, 50 mL of 52.0% Baijiu (a traditional Chinese distilled liquor) contains 20.5 grams of pure alcohol; a 500 mL can of 3.3% beer contains 13.0 grams of pure alcohol; a 140 mL glass of 13.0% wine contains 14.4 grams of pure alcohol; and a 350 mL bottle of 15.0% Chinese yellow wine contains 41.4 grams of pure alcohol.

#### 2.2.3 Adherence to ART

Adherence to ART was evaluated using the Center for Adherence Support Evaluation (CASE) Index, a 3-item tool designed to measure ART adherence. The composite score ranges from 3 to 16, with higher scores indicating better adherence. According to CASE Index guidelines, ART non-adherence is defined as a score of 10 or lower (23). The Chinese version of the CASE Index has been validated and demonstrated good reliability and validity (24). In this study, the CASE Index exhibited high internal consistency, with a Cronbach’s alpha of 0.944.

#### 2.2.4 Sexual behaviors

MSM participants were also surveyed about their sexual behaviors, including sexual role preference (insertive, receptive, or versatile), age at initiation of anal sex (<18 years or ≥18 years), number of male sex partners (<2 or ≥2), and involvement in commercial sex (defined as engaging in sexual activity in exchange for money, drugs, goods, or accommodation, yes/no), casual sex (defined as engaging in sexual activity with other males apart from their spouse, fixed boyfriend, lover or commercial sex partners, yes/no), and group sex (defined as having 2 or more sex partners during a single sexual encounter, yes/no) in the previous 6 months. Additionally, they were asked whether they engaged in condomless internal ejaculation (CIE) during anal sex in the previous 6 months (yes/no).

### 2.3 Data analysis

The survey data were exported from the Wenjuanxing platform and analyzed using SPSS version 22.0 (IBM Corp., Armonk, NY, USA). Categorical variables were summarized as frequencies and percentages, and group comparisons were conducted using Pearson’s chi-squared test. Continuous variables were expressed as medians and interquartile ranges (IQR), with group comparisons performed using the Mann–Whitney U test. To identify factors associated with alcohol consumption and to examine the effects of alcohol consumption patterns on ART adherence and sexual risk-taking behaviors among MSM living with HIV, binary logistic regression models were utilized. Variables with a *p*-value less than 0.10 in univariate logistic regression analyses were included in the multivariate logistic regression model. Factors with a *p*-value less than 0.05 in the multivariate analysis were retained in the final regression model. Odds ratios (ORs) with 95% confidence intervals (CIs) were calculated to determine the strength of associations. Prior to conducting the multivariate logistic regression, multicollinearity among the independent variables was assessed by examining pairwise correlation coefficients. All correlation coefficients were below 0.6, indicating that multicollinearity was not a concern in our analysis.

## 3 Results

### 3.1 Participant characteristics

A total of 1,550 MSM living with HIV were initially invited to participate in this study. After excluding 10 MSM (0.6%) who declined to participate and 39 MSM (2.5%) who did not complete the online survey, a final sample of 1,501 participants was included in the analysis. The majority of participants were aged 26 years or older (85.9%), unmarried, divorced, or widowed (81.9%), and non-migrants (79.3%). A significant proportion of participants had at least a college education (77.3%), were employed full-time (78.3%), and had an individual monthly income exceeding 5,000 CNY (61.0%). Over half (51.9%) had been diagnosed with HIV for 5 years or longer. Most participants (97.9%) were currently receiving ART, with 92.2% demonstrating good adherence. Among the participants, 84.4% had an HIV viral load of <1,000 copies/mL. In terms of mental health, 40.8% of participants reported experiencing depressive symptoms, and 29.6% reported anxiety symptoms. The median HIV-related stigma score was 26.0 (IQR: 21.0–32.0) (See Table 1).

**Table 1:** Sociodemographic characteristics, antiretroviral treatment adherence, and mental health among MSM living with HIV in Chongqing, stratified by alcohol consumption patterns (*N* = 1,501).

Variables	*n* (%)	Alcohol use (+)		Hazardous drinking (+)		Binge drinking (+)	
		*n* (%)	*p*	*n* (%)	*p*	*n* (%)	*p*
Age (years)							
18–25	211 (14.1)	129 (61.1)	0.848	72 (34.1)	<0.001	93 (44.1)	0.902
26–35	718 (47.8)	435 (60.6)		191 (26.6)		316 (44.0)	
>35	572 (38.1)	339 (59.3)		110 (19.2)		245 (42.8)	
Current marital status							
Married, cohabiting with females	271 (18.1)	179 (66.1)	0.029	65 (24.0)	0.716	127 (46.9)	0.227
Unmarried, divorced or widowed	1,230 (81.9)	724 (58.9)		308 (25.0)		527 (42.8)	
Migrant							
Yes	311 (20.7)	213 (68.5)	0.001	107 (34.4)	<0.001	171 (55.0)	<0.001
No	1,190 (79.3)	690 (58.0)		266 (22.4)		483 (40.6)	
Education attainment							
Senior high school and below	340 (22.7)	239 (70.3)	<0.001	114 (33.5)	<0.001	181 (53.2)	<0.001
College and above	1,161 (77.3)	664 (57.2)		259 (22.3)		473 (40.7)	
Employment status							
Full-time	1,176 (78.3)	691 (58.8)	0.035	273 (23.2)	0.005	491 (41.8)	0.007
Part-time or other	325 (21.7)	212 (65.2)		100 (30.8)		163 (50.2)	
Individual monthly income (CNY)							
≤5,000	585 (39.0)	351 (60.0)	0.919	130 (22.2)	0.060	246 (42.1)	0.343
>5,000	916 (61.0)	552 (60.3)		243 (26.5)		408 (44.5)	
Duration since HIV diagnosis (years)							
≤1	154 (10.3)	77 (50.0)	0.006	33 (21.4)	<0.001	47 (30.5)	<0.001
2–4	568 (37.8)	363 (63.9)		175 (30.8)		273 (48.1)	
≥5	779 (51.9)	463 (59.4)		165 (21.2)		334 (42.9)	
On ART							
Yes	1,469 (97.9)	888 (60.4)	0.121	366 (24.9)	0.694	642 (43.7)	0.484
No	32 (2.1)	15 (46.9)		7 (21.9)		12 (37.5)	
Adherence to ART							
Yes	1,355 (92.2)	793 (58.5)	<0.001	292 (21.5)	<0.001	556 (41.0)	<0.001
No	114 (7.8)	95 (83.3)		74 (64.9)		86 (75.4)	
HIV viral load (copies/mL)							
<1,000	1,267 (84.4)	757 (59.7)	0.015	310 (24.5)	0.689	551 (43.5)	0.524
≥1,000	56 (3.7)	26 (46.4)		16 (28.6)		21 (37.5)	
Not tested	178 (11.9)	120 (67.4)		47 (26.4)		82 (46.1)	
Depressive symptoms							
Yes	612 (40.8)	400 (65.4)	0.001	169 (27.6)	0.040	290 (47.4)	0.013
No	889 (59.2)	503 (56.6)		204 (22.9)		364 (40.9)	
Anxiety symptoms							
Yes	445 (29.6)	273 (61.3)	0.542	112 (25.2)	0.853	195 (43.8)	0.899
No	1,056 (70.4)	630 (59.7)		261 (24.7)		459 (43.5)	
Perceived HIV-related stigma [Median (IQR)]	26.0 (21.0–32.0)	27.0 (22.0–31.0)	0.527	27.0 (22.5–32.0)	0.017	27.0 (22.0–32.0)	0.257

HIV, human immunodeficiency virus; MSM, men who have sex with men; CNY, Chinese Yuan; ART, antiretroviral treatment; IQR, interquartile range.

### 3.2 Alcohol consumption

Regarding alcohol consumption, alcohol use, hazardous drinking, and binge drinking were reported by 60.2, 24.9 and 43.6% of participants, respectively. Among the 903 participants who reported alcohol consumption, 62.5% consumed alcohol once a month or less, and 29.0% consumed alcohol 2–4 times per month. In terms of alcohol intake, 38.6% consumed 1–2 standard drinks on a typical day, and 48.4% consumed 3–4 standard drinks on a typical day. As for binge drinking frequency, 44.6% of participants reported binge drinking less than once a month, 23.8% reported binge drinking monthly, and 4.0% reported binge drinking weekly or more.

### 3.3 Recreational drug use and sexual behaviors

Of the 1,501 participants, 32.5% reported recreational drug use in the previous 6 months, with poppers being the most commonly used substance (32.4%). In terms of sexual behaviors, 13.4% reported initiating anal sex before the age of 18, and 34.9% preferred insertive sexual role. In the previous 6 months, 59.2% reported having at least two male sexual partners, and 9.3% participated in group sex. Additionally, 62.0 and 12.5% of participants reported engaging in casual sex and commercial sex, respectively. Furthermore, 34.2% reported practicing CIE in the previous 6 months (See Table 2).

**Table 2:** Behavioral characteristics among MSM living with HIV in Chongqing, stratified by alcohol consumption patterns (*N* = 1,501).

Variables	*n* (%)	Alcohol use (+)		Hazardous drinking (+)		Binge drinking (+)	
		*n* (%)	*p*	*n* (%)	*p*	*n* (%)	*p*
Recreational drug use in the past 6 months							
Yes	488 (32.5)	356 (73.0)	<0.001	185 (37.9)	<0.001	306 (62.7)	<0.001
No	1,013 (67.5)	547 (54.0)		188 (18.6)		348 (34.4)	
Initiating anal sex before 18 years							
Yes	201 (13.4)	138 (68.7)	0.008	86 (42.8)	<0.001	113 (56.2)	<0.001
No	1,300 (86.6)	765 (58.8)		287 (22.1)		541 (41.6)	
Sexual role preference during anal sex							
Insertive	524 (34.9)	341 (65.1)	<0.001	146 (27.9)	0.007	274 (52.3)	<0.001
Receptive	481 (32.1)	245 (50.9)		95 (19.8)		148 (30.8)	
Versatile	496 (33.0)	317 (63.9)		132 (26.6)		232 (46.8)	
Having multiple male sex partners in the past 6 months							
Yes	889 (59.2)	592 (66.6)	<0.001	268 (30.1)	<0.001	461 (51.9)	<0.001
No	612 (40.8)	311 (50.8)		105 (17.2)		193 (31.5)	
Engaging in casual sex in the past 6 months							
Yes	930 (62.0)	598 (64.3)	<0.001	263 (28.3)	<0.001	464 (49.9)	<0.001
No	571 (38.0)	305 (53.4)		110 (19.3)		190 (33.3)	
Engaging in commercial sex in the past 6 months							
Yes	187 (12.5)	159 (85.0)	<0.001	110 (58.8)	<0.001	149 (79.7)	<0.001
No	1,314 (87.5)	744 (56.6)		263 (20.0)		505 (38.4)	
Participating in group sex in the past 6 months							
Yes	139 (9.3)	109 (78.4)	<0.001	64 (46.0)	<0.001	99 (71.2)	<0.001
No	1,362 (90.7)	794 (58.3)		309 (22.7)		555 (40.7)	
Practicing CIE during anal sex in the past 6 months							
Yes	514 (34.2)	339 (66.0)	0.001	166 (32.3)	<0.001	275 (53.5)	<0.001
No	987 (65.8)	564 (57.1)		207 (21.0)		379 (38.4)	

HIV, human immunodeficiency virus; MSM, men who have sex with men; ART, antiretroviral treatment; IQR, interquartile range; CIE, Condomless internal ejaculation.

### 3.4 Factors associated with alcohol consumption

Multivariate logistic regression analysis identified several factors significantly associated with alcohol consumption patterns. Being a migrant (adjusted odds ratio [aOR] = 1.42, 95% CI: 1.07–1.88; aOR = 1.41, 95% CI: 1.05–1.91; aOR = 1.54, 95% CI: 1.17–2.02), having lower educational attainment (aOR = 1.51, 95% CI: 1.14–1.99; aOR = 1.99, 95% CI: 1.45–2.73; aOR = 1.33, 95% CI: 1.01–1.74), duration since HIV diagnosis ≥2 years (aOR = 1.94, 95% CI: 1.31–2.87; aOR = 2.39, 95% CI: 1.43–3.98; aOR = 2.49, 95% CI: 1.62–3.83) and ≥5 years (aOR = 1.57, 95% CI: 1.06–2.31; aOR = 1.78, 95% CI: 1.03–3.08; aOR = 2.04, 95% CI: 1.33–3.13), preferring insertive sexual role (aOR = 1.75, 95% CI: 1.33–2.31; aOR = 1.69, 95% CI: 1.20–2.37; aOR = 2.50, 95% CI: 1.88–3.33), and recreational drug use (aOR = 1.83, 95% CI: 1.43–2.36; aOR = 2.28, 95% CI: 1.74–3.00; aOR = 2.63, 95% CI: 2.06–3.35) were associated with all three alcohol consumption patterns. Preferring versatile sexual role (aOR = 1.60, 95% CI: 1.22–2.09; aOR = 1.92, 95% CI: 1.45–2.54) was associated with alcohol use and binge drinking. Initiating anal sex before the age of 18 (aOR = 1.87, 95% CI: 1.27–2.76; aOR = 1.73, 95% CI: 1.22–2.46) was associated with hazardous drinking and binge drinking. Participants aged 18–25 years (aOR = 2.66, 95% CI: 1.54–4.61) and 26–35 years (aOR = 1.51, 95% CI: 1.10–2.08), with higher individual monthly income (aOR = 2.11, 95% CI: 1.52–2.93), and perceiving higher levels of HIV-related stigma (aOR = 1.02, 95% CI: 1.00–1.04) were more likely to report hazardous drinking. Additionally, being married or cohabiting with a female partner (aOR = 1.36, 95% CI: 1.02–1.82) and experiencing depressive symptoms (aOR = 1.36, 95% CI: 1.07–1.72) were specifically associated with alcohol use (See Table 3).

**Table 3:** Factors associated with alcohol consumption patterns among MSM living with HIV in Chongqing.

Factors	Alcohol use		Hazardous drinking		Binge drinking	
	OR (95% CI)	aOR (95% CI)	OR (95% CI)	aOR (95% CI)	OR (95% CI)	aOR (95% CI)
Age (years)						
>35	1.00		1.00	1.00	1.00	
26–35	1.06 (0.84–1.32)		1.52 (1.17–1.99)^*^	1.51 (1.10–2.08)^*^	1.05 (0.84–1.31)	
18–25	1.08 (0.78–1.49)		2.18 (1.53–3.10)^**^	2.66 (1.54–4.61)^*^	1.05 (0.77–1.45)	
Current marital status						
Unmarried, divorced or widowed	1.00	1.00	1.00		1.00	
Married, cohabiting	1.36 (1.03–1.79)^*^	1.36 (1.02–1.82)^*^	0.95 (0.70–1.28)		1.18 (0.90–1.53)	
Migration status						
No	1.00	1.00	1.00	1.00	1.00	1.00
Yes	1.58 (1.21–2.05)^*^	1.42 (1.07–1.88)^*^	1.82 (1.39–2.39)^**^	1.41 (1.05–1.91)^*^	1.79 (1.39–2.30)^**^	1.54 (1.17–2.02)^*^
Education attainment						
College and above	1.00	1.00	1.00	1.00	1.00	1.00
Senior high school and below	1.77 (1.37–2.30)^**^	1.51 (1.14–1.99)^*^	1.76 (1.35–2.29)^**^	1.99 (1.45–2.73)^**^	1.66 (1.30–2.11)^**^	1.33 (1.01–1.74)^*^
Employment status						
Full-time	1.00	1.00	1.00	1.00	1.00	1.00
Part-time or other	1.32 (1.02–1.70)^*^	1.10 (0.83–1.48)	1.47 (1.12–1.93)^*^	1.21 (0.83–1.75)	1.40 (1.10–1.80)^*^	1.16 (0.87–1.56)
Individual monthly income (CNY)						
≤5,000	1.00		1.00	1.00	1.00	
>5,000	1.01 (0.82–1.25)		1.26 (0.99–1.61)	2.11 (1.52–2.93)^**^	1.11 (0.90–1.37)	
Sexual role preference during anal sex						
Receptive	1.00	1.00	1.00	1.00	1.00	1.00
Versatile	1.71 (1.32–2.20)^**^	1.60 (1.22–2.09)^*^	1.47 (1.09–1.99)^*^	1.38 (0.99–1.92)	1.98 (1.52–2.570)^**^	1.92 (1.45–2.54)^**^
Insertive	1.80 (1.39–2.31)^**^	1.75 (1.33–2.31)^**^	1.57 (1.17–2.11)^*^	1.69 (1.20–2.37)^*^	2.47 (1.90–3.19)^**^	2.50 (1.88–3.33)^**^
Duration since HIV diagnosis (years)						
≤1	1.00	1.00	1.00	1.00	1.00	1.00
2–4	1.77 (1.24–2.54)^*^	1.94 (1.31–2.87)^*^	1.63 (1.07–2.50)^*^	2.39 (1.43–3.98)^*^	2.11 (1.44–3.08)^**^	2.49 (1.62–3.83)^**^
≥5	1.47 (1.04–2.07)^*^	1.57 (1.06–2.31)^*^	0.99 (0.65–1.50)	1.78 (1.03–3.08)^*^	1.71 (1.18–2.48)^*^	2.04 (1.33–3.13)^*^
Depressive symptoms						
No	1.00	1.00	1.00	1.00	1.00	1.00
Yes	1.45 (1.17–1.79)^*^	1.36 (1.07–1.72)^*^	1.28 (1.01–1.62)^*^	1.04 (0.78–1.40)	1.30 (1.06–1.60)^*^	1.15 (0.90–1.46)
Anxiety symptoms						
No	1.00		1.00		1.00	
Yes	1.07 (0.86–1.35)		1.02 (0.79–1.32)		1.02 (0.81–1.27)	
Perceived HIV-related stigma	1.00 (0.99–1.02)		1.02 (1.00–1.03)^*^	1.02 (1.00–1.04)^*^	1.01 (0.99–1.02)	
Initiating anal sex before 18 years						
No	1.00	1.00	1.00	1.00	1.00	1.00
Yes	1.53 (1.12–2.11)^*^	1.40 (0.98–2.00)	2.64 (1.94–3.59)^**^	1.87 (1.27–2.76)^*^	1.80 (1.34–2.43)^**^	1.73 (1.22–2.46)^*^
Recreational drug use in the past 6 months						
No	1.00	1.00	1.00	1.00	1.00	1.00
Yes	2.30 (1.82–2.91)^**^	1.83 (1.43–2.36)^**^	2.68 (2.10–3.41)^**^	2.28 (1.74–3.00)^**^	3.21 (2.57–4.02)^**^	2.63 (2.06–3.35)^**^

HIV, human immunodeficiency virus; MSM, men who have sex with men; OR, odds ratio; CI, confidence interval; aOR, adjusted odds ratio; CNY, Chinese Yuan.

*p < 0.05; **p < 0.001.

### 3.5 Associations of alcohol consumption patterns with ART adherence and sexual risk-taking behaviors

A total of 18 multivariate logistic regression models were constructed, with ART adherence or sexual risk-taking behaviors as the dependent variables and various alcohol consumption patterns as the independent variables. These models were adjusted for other demographic and behavioral variables with *p*-values less than 0.10. The results revealed that participants who consumed alcohol, engaged in hazardous drinking, or engaged in binge drinking were more likely to report ART non-adherence (aOR = 1.89, 95% CI: 1.08–3.30; aOR = 3.43, 95% CI: 2.11–5.58; aOR = 2.17, 95% CI: 1.30–3.62), have multiple sexual partners (aOR = 1.62, 95% CI: 1.27–2.06; aOR = 1.49, 95% CI: 1.11–1.99; aOR = 1.65, 95% CI: 1.29–2.11), engage in commercial sex (aOR = 2.50, 95% CI: 1.56–3.99; aOR = 3.21, 95% CI: 2.16–4.75; aOR = 3.11, 95% CI: 2.03–4.75), and participate in group sex (aOR = 1.73, 95% CI: 1.05–2.83; aOR = 1.79, 95% CI: 1.14–2.80; aOR = 1.77, 95% CI: 1.12–2.80). Alcohol consumption and binge drinking were also associated with a greater likelihood of engaging in casual sex (aOR = 1.31, 95% CI: 1.03–1.66; aOR = 1.52, 95% CI: 1.18–1.95). Hazardous drinking and binge drinking were additionally linked to higher odds of practicing CIE during anal sex (aOR = 1.37, 95% CI: 1.03–1.83; aOR = 1.44, 95% CI: 1.11–1.86) (See Table 4).

**Table 4:** Associations of alcohol consumption patterns with ART adherence and sexual risk-taking behaviors among MSM living with HIV in Chongqing.

Alcohol consumption patterns	ART non-adherence^a^	Having multiple sex partners^b^	Engaging in commercial sex^c^	Engaging in casual sex^d^	Participating in group sex^e^	Practicing CIE during anal sex^f^
	aOR (95% CI)	aOR (95% CI)	aOR (95% CI)	aOR (95% CI)	aOR (95% CI)	aOR (95% CI)
Alcohol use						
No	1.00	1.00	1.00	1.00	1.00	1.00
Yes	1.89 (1.08–3.30)^*^	1.62 (1.27–2.06)^**^	2.50 (1.56–3.99)^**^	1.31 (1.03–1.66)^*^	1.73 (1.05–2.83)^*^	1.17 (0.90–1.51)
Hazardous drinking						
No	1.00	1.00	1.00	1.00	1.00	1.00
Yes	3.43 (2.11–5.58)^**^	1.49 (1.11–1.99)^*^	3.21 (2.16–4.75)^**^	1.32 (0.98–1.77)	1.79 (1.14–2.80)^*^	1.37 (1.03–1.83)^*^
Binge drinking						
No	1.00	1.00	1.00	1.00	1.00	1.00
Yes	2.17 (1.30–3.62)^*^	1.65 (1.29–2.11)^**^	3.11 (2.03–4.75)^**^	1.52 (1.18–1.95)^*^	1.77 (1.12–2.80)^*^	1.44 (1.11–1.86)^*^

ART, antiretroviral treatment; HIV, human immunodeficiency virus; MSM, men who have sex with men; aOR, adjusted odds ratio; CI, confidence interval; CIE, Condomless internal ejaculation.

^*^p < 0.05; ^**^p < 0.001.

a Adjusted for age, current marital status, migrant, education attainment, employment status, individual monthly income, sexual role preference, depressive symptoms, anxiety symptoms, perceived HIV-related stigma, initiating anal sex before 18 years, recreational drug use and HIV viral load.

b Adjusted for age, current marital status, migrant, education attainment, employment status, individual monthly income, sexual role preference, depressive symptoms, anxiety symptoms, initiating anal sex before 18 years and recreational drug use.

c Adjusted for age, current marital status, migrant, education attainment, employment status, individual monthly income, sexual role preference, initiating anal sex before 18 years and recreational drug use.

d Adjusted for age, current marital status, migrant, education attainment, employment status, individual monthly income, depressive symptoms, anxiety symptoms, perceived HIV-related stigma, initiating anal sex before 18 years and recreational drug use.

e Adjusted for age, current marital status, migrant, employment status, individual monthly income, duration since HIV diagnosis, depressive symptoms, anxiety symptoms, perceived HIV-related stigma, initiating anal sex before 18 years and recreational drug use.

f Adjusted for age, current marital status, migrant, education attainment, employment status, individual monthly income, sexual role preference, duration since HIV diagnosis, depressive symptoms, anxiety symptoms, perceived HIV-related stigma, initiating anal sex before 18 years and recreational drug use.

## 4 Discussion

This study is among the first investigations in China to examine associations between alcohol consumption patterns, ART adherence, and sexual risk-taking among MSM living with HIV. Our findings indicate that alcohol consumption is prevalent among MSM living with HIV in Chongqing, with 60.2% reporting alcohol use, 24.9% engaging in hazardous drinking, and 43.6% reporting binge drinking. These estimates are comparable to reports among general MSM in Beijing (56.1–57.9%) (2, 6) and an online survey (62.1%) (25). They also appear higher than some estimates among general MSM in Chongqing (23.0%) (12) and Sichuan Province (44.0%) (14), and among men living with HIV in the general population (35.2%) (26). Because sampling frames, measures, and time periods differ across studies, these comparisons should be interpreted cautiously. Nonetheless, the high prevalence observed in our Chongqing sample warrants close attention.

Several social and demographic factors, such as migration status, educational attainment, age, and individual monthly income, were identified as correlates of alcohol consumption patterns among MSM living with HIV. Similar results have been found in prior studies (2, 6, 14). Migrant populations often face various social and psychological challenges, such as unstable living conditions, lower access to health services, and discrimination, which can contribute to psychological distress and health-risk behaviors, including alcohol use (27, 28). Additionally, MSM with lower educational attainment were found to be at higher risk for alcohol use, hazardous drinking, and binge drinking. This may reflect limited health literacy and a higher likelihood of using alcohol as a coping mechanism for the psychological burdens associated with living with HIV. Younger MSM were more inclined to engage in hazardous drinking, possibly due to increased social activity and participation in nightlife or party scenes where alcohol consumption is more common. Peer influence in these settings may reinforce risky drinking patterns. Individual monthly income was found to be positively associated with hazardous drinking among MSM living with HIV, suggesting that individuals with higher income might have greater financial means to purchase alcohol, thereby facilitating higher consumption levels. Additionally, higher income may be linked to social contexts where alcohol consumption is prevalent, further promoting hazardous drinking behaviors.

Marital status and depressive symptoms were associated with alcohol use, and higher levels of HIV-related stigma were associated with hazardous drinking. Given the stigma surrounding homosexuality and the traditional expectations of marriage and reproduction in China, some MSM may marry or cohabit with women, which can lead to involuntary heterosexual encounters (29). In these situations, alcohol consumption may serve as a coping mechanism for emotional or identity-related conflicts (25). The results of this study indicated that MSM living with HIV faced significant challenges related to HIV-related stigma and depressive symptoms, and those who perceived higher levels of HIV-related stigma and experienced depressive symptoms were more likely to consume alcohol. This aligns with prior research suggesting that individuals living with HIV may turn to alcohol as a means of coping with psychological stress caused by societal discrimination and HIV stigma (13, 26, 30).

In addition to these factors, our study found that sexual role preferences and the duration since HIV diagnosis were associated with all alcohol consumption patterns. Globally and in China, alcohol consumption is more prevalent among men (1, 31), suggesting a possible link between gender identity and alcohol consumption. Among MSM, those who prefer insertive or versatile sexual roles may be more likely to identify with traditional masculine traits, such as dominance and assertiveness (32). These traits, in turn, could promote alcohol consumption in social contexts where they are valued and reinforced (33). Moreover, MSM living with HIV for two or more years were more likely to report alcohol use, hazardous drinking, and binge drinking. This pattern may be due to individuals initially adhering strictly to medical advice to avoid alcohol during the early stages of treatment (34). As their health stabilizes, they may experience reduced self-control over alcohol use, leading to higher odds of alcohol consumption.

Our findings also indicate that an earlier age at first anal intercourse and recreational drug use are associated with higher levels of alcohol consumption among MSM living with HIV. Individuals who initiate anal sex at a younger age may exhibit greater sensation-seeking behavior (35), making them more susceptible to engaging in risky behaviors, including substance abuse (25). Alcohol and recreational drugs are frequently used in sexual settings among MSM (36, 37), and the combination of alcohol with stimulants like methamphetamine may mask the depressant effects of alcohol. This can make it more difficult for individuals to assess their level of intoxication, thereby increasing the risk of hazardous drinking and binge drinking.

Previous studies have consistently shown that alcohol use is associated with poorer ART adherence among individuals living with HIV (15–17). Our findings further support this by showing that MSM living with HIV who consumed alcohol, particularly those engaging in hazardous drinking or binge drinking, were more likely to demonstrate higher levels of ART non-adherence. This is likely linked to the negative cognitive and behavioral effects of alcohol. Alcohol has been shown to impair cognitive function and reduce health awareness (38), which can weaken an individual’s ability to manage their treatment regimen and adhere to medical advice. Additionally, some studies suggest that individuals might intentionally avoid taking ART while consuming alcohol due to the belief that combining alcohol with ART is harmful or renders the medication ineffective (39). Consequently, non-adherence to ART can lead to insufficient viral suppression, decreased immune function, and accelerated disease progression.

Our findings align with existing evidence underscoring the robust association between alcohol consumption and sexual risk-taking behaviors (2–7). MSM living with HIV who consumed alcohol, particularly those who engaged in hazardous drinking or binge drinking, were more likely to have multiple sexual partners, engage in commercial sex, and participate in group sex. Alcohol consumption, especially binge drinking, was associated with higher rates of casual sexual encounters. This relationship may be partially explained by the spatial overlap between alcohol consumption environments and settings where MSM meet potential sexual partners (25). Venues that serve alcohol, such as bars and nightclubs, are common social spaces where MSM seek sexual partners and clients. Additionally, alcohol is often used to boost confidence and promote relaxation (40). However, these effects are coupled with increased sexual desire, reduced inhibition, and cognitive impairments (4–6). As a result, MSM may engage in riskier sexual behaviors, such as having multiple partners, participating in casual sex, or engaging in commercial or group sex, while under the influence of alcohol.

Furthermore, it is well-documented that alcohol consumption is associated with higher odds of condomless anal sex, a finding that is confirmed in our study (2, 41–42). As established in the literature, excessive alcohol consumption and acute intoxication directly impair cognitive functions related to judgment, decision-making, inhibitory control, and risk perception (2, 41–42). These deficits significantly compromise an individual’s ability to consistently negotiate condom use or adhere to safer sex intentions in the moment. This provides a possible neurocognitive pathway that explains the elevated risk of CIE observed among MSM with hazardous and binge drinking behaviors within our sample. In addition, the effects of alcohol on sexual risk-taking behavior should also be considered within the specific socio-cultural context of China. Alcohol consumption, particularly binge drinking, is often deeply embedded in social and sexual interactions within this population (25). Collectivist drinking behaviors, such as peer pressure to “ganbei” (drinking the entire glass) during group gatherings (43), and the frequent consumption of alcohol in venues associated with sexual opportunities (e.g., bars, clubs, KTVs, or private parties following social events) (25) create environments where heavy episodic drinking is common and often expected. These sexually charged or socially pressured settings, combined with the acute neurocognitive effects of intoxication, create a high-risk synergy. This suggests that alcohol not only directly influences sexual behavior through its pharmacological effects on cognition but also enhances the occurrence of unprotected sexual activities through its embeddedness in specific high-risk social contexts in China.

Several limitations of this study should be considered when interpreting our findings. First, given challenges in reaching MSM living with HIV, we recruited participants through two CBOs. This approach may have excluded individuals who are less engaged with services, and participants recruited via CBOs might differ from those not connected to such organizations. In our setting, HIV-related stigma remains substantial, and many MSM living with HIV keep their status confidential. Therefore, we used convenience sampling rather than respondent-driven sampling (RDS) for feasibility and ethical reasons. The sample was skewed toward older, non-migrant, college-educated, employed, and higher-income participants, who also reported better ART adherence and higher viral suppression. These factors limit the generalizability of our findings beyond MSM living with HIV engaged with CBO services in Chongqing and to other regions of China. Future studies should consider probability sampling methods (e.g., RDS) to improve the representativeness of the sample and address these limitations. Second, data on HIV status, viral load, alcohol consumption, ART adherence, sexual behaviors and drug use were collected through self-administered questionnaires, and thus recall bias may exist. Third, participants were asked about their drug use and sexual risk-taking behaviors (casual sex, commercial sex, group sex and CIE), which are sensitive topics in China. Given the social stigma surrounding these behaviors, participants may have concealed certain information due to social desirability bias, potentially leading to underreporting or underestimation of the true prevalence and associations. To mitigate this risk, we used an anonymous, self-administered online questionnaire, collected no personally identifiable information, and ensured that CBO staff could not access individual responses. Participants were also informed that their data would be used solely for research purposes to encourage candid reporting. Fourth, this study explored the associations between alcohol consumption patterns and sexual risk-taking behaviors. However, the frequency of risk-taking behaviors and the HIV status of participants’ partners were not considered in this analysis. Future research should collect data on the frequency of risk-taking behaviors and investigate the association between alcohol consumption and CIE with HIV-negative partners to better assess the risk of HIV transmission associated with alcohol use. Fifth, in this study, ART adherence was assessed solely with the CASE Index. We incorporated viral load into the adherence analysis and observed higher odds of CASE-defined non-adherence among participants with viral load ≥1,000 copies/mL. The result provides some support for the utility of the CASE Index as a screening measure. Nonetheless, future studies should combine validated self-report instruments with objective adherence metrics (e.g., pharmacy refill records and pill counts) and viral load measurements to strengthen measurement validity. Additionally, this study did not collect laboratory data on participants’ HIV viral load, limiting our ability to assess how alcohol-related ART non-adherence might influence clinical outcomes. Future studies incorporating laboratory measures would provide a more comprehensive understanding of this relationship. Furthermore, we used the AUDIT-C to assess alcohol consumption patterns but did not gather information on alcohol use specifically in relation to sexual activity. Future research should include detailed data on alcohol consumption in sexual contexts to better assess its effects on sexual risk behaviors. Finally, given the cross-sectional design of this study, we cannot infer causality from the observed associations between alcohol consumption patterns, ART adherence, and sexual risk-taking behaviors. Longitudinal studies are needed to establish causal relationships and determine the directionality of these associations.

## 5 Conclusion

In this convenience sample of MSM living with HIV in Chongqing, alcohol consumption, particularly hazardous and binge drinking, is common and is associated with higher odds of ART non-adherence and sexual risk-taking behaviors. Public health strategies in Chongqing and similar settings may benefit from screening and interventions to address alcohol consumption among MSM living with HIV. Correlates identified in this sample that could help tailor programs include lower educational attainment, higher monthly income, longer duration since HIV diagnosis, HIV-related stigma, depressive symptoms, insertive sexual role preference, younger age, earlier initiation of anal sex, and recreational drug use. Integrating alcohol reduction components into ART support and sexual health services for MSM living with HIV in Chongqing and similar settings may improve individual health outcomes and reduce transmission risks.

## Data Availability

The raw data supporting the conclusions of this article will be made available by the authors, without undue reservation.
