# Supplementary material for: Alcohol consumption patterns and risk of HIV transmission among men who have sex with men living with HIV in Chongqing, southwestern China
Source: Front Public Health. 2025 Oct 15;13:1629368. doi: 10.3389/fpubh.2025.1629368 (PMC12568656; doi:10.3389/fpubh.2025.1629368)
Supplement: Supplementary file 1 [file Supplementary_file_1.doc]

| TABLE S1 Associations of ART adherence with HIV viral load among MSM living with HIV in Chongqing | | | | | | | |
| --- | --- | --- | --- | --- | --- | --- | --- |
|  | HIV viral load <1000 copies/mL | | | | | | |
|  | *OR* (*95%CI*) |  | *aOR* (*95%CI*)^a^ |  | *aOR* (*95%CI*)^b^ |  | *aOR* (*95%CI*)^c^ |
| **Adherence to ART** |  |  |  |  |  |  |  |
| No | 1.00 |  | 1.00 |  | 1.00 |  | 1.00 |
| Yes | 3.81(2.52-5.77)** |  | 1.85(1.06-3.22)* |  | 2.03(1.15-2.59)* |  | 1.94(1.11-3.39)* |
| ART, antiretroviral treatment; HIV, human immunodeficiency virus; MSM, men who have sex with men; OR, odds ratio; aOR, adjusted odds ratio; CI, confidence interval | | | | | | | |
| *p-value < 0.05; **p-value < 0.001 | | | | | | | |
| ^a^adjusted for adjusted for age, current marital status, migrant, education attainment, employment status, individual monthly income, sexual role preference, duration since HIV diagnosis, depressive symptoms, anxiety symptoms, perceived HIV-related stigma, initiating anal sex before 18 years, recreational drug use, and alcohol use. | | | | | | | |
| ^b^adjusted for age, current marital status, migrant, education attainment, employment status, individual monthly income, sexual role preference, duration since HIV diagnosis, depressive symptoms, anxiety symptoms, perceived HIV-related stigma, initiating anal sex before 18 years, recreational drug use, and hazardous drinking. | | | | | | | |
| ^c^adjusted for age, current marital status, migrant, education attainment, employment status, individual monthly income, sexual role preference, duration since HIV diagnosis, depressive symptoms, anxiety symptoms, perceived HIV-related stigma, initiating anal sex before 18 years, recreational drug use, and binge drinking. | | | | | | | |

| TABLE S2 Univariate analysis on the associations of alcohol consumption patterns with ART adherence and sexual risk-taking behaviors among MSM living with HIV in Chongqing | | | | | | | | | | | |
| --- | --- | --- | --- | --- | --- | --- | --- | --- | --- | --- | --- |
| Alcohol consumption patterns | ART non-adherence |  | Having multiple sex partners |  | Engaging in commercial sex |  | Engaging in casual sex |  | Participating in group sex |  | Practicing CIE during anal sex |
|  | *OR* (95%CI) |  | *OR* (95%CI) |  | *OR* (95%CI) |  | *OR* (95%CI) |  | *OR* (95%CI) |  | *OR* (95%CI) |
| Alcohol use |  |  |  |  |  |  |  |  |  |  |  |
| No | 1.00 |  | 1.00 |  | 1.00 |  | 1.00 |  | 1.00 |  | 1.00 |
| Yes | 3.54(2.14-5.87)^**^ |  | 1.93(1.56-2.38)^**^ |  | 4.35(2.87-6.60)^**^ |  | 1.57(1.27-1.94)^**^ |  | 2.60(1.71-3.95)^**^ |  | 1.45(1.16-1.81)^*^ |
| Hazardous drinking |  |  |  |  |  |  |  |  |  |  |  |
| No | 1.00 |  | 1.00 |  | 1.00 |  | 1.00 |  | 1.00 |  | 1.00 |
| Yes | 6.74(4.49-10.11)^**^ |  | 2.08(1.62-2.69)^**^ |  | 5.71(4.14-7.87)^**^ |  | 1.65(1.28-2.13)^**^ |  | 2.91(2.04-4.16)^**^ |  | 1.80(1.41-2.29)^**^ |
| Binge drinking |  |  |  |  |  |  |  |  |  |  |  |
| No | 1.00 |  | 1.00 |  | 1.00 |  | 1.00 |  | 1.00 |  | 1.00 |
| Yes | 4.41(2.84-6.85)^**^ |  | 2.34(1.89-2.90)^**^ |  | 6.28(4.33-9.12)^**^ |  | 2.00(1.61-2.48)^**^ |  | 3.60(2.45-5.28)^**^ |  | 1.85(1.49-2.29)^**^ |
| ART, antiretroviral treatment; HIV, human immunodeficiency virus; MSM, men who have sex with men; aOR, adjusted odds ratio; CI, confidence interval; CIE, Condomless internal ejaculation | | | | | | | | | | | |
| ^*^P < 0.05; ^**^P < 0.001 | | | | | | | | | | | |

| TABLE S3 Associations of alcohol consumption patterns with ART adherence and sexual risk-taking behaviors among MSM living with HIV in Chongqing | | | | | | | | | | | |
| --- | --- | --- | --- | --- | --- | --- | --- | --- | --- | --- | --- |
| Alcohol consumption patterns | ART non-adherence |  | Having multiple sex partners |  | Engaging in commercial sex |  | Engaging in casual sex |  | Participating in group sex |  | Practicing CIE during anal sex |
|  | *aOR* (95%CI) |  | *aOR* (95%CI) |  | *aOR* (95%CI) |  | *aOR* (95%CI) |  | *aOR* (95%CI) |  | *aOR* (95%CI) |
| Alcohol use |  |  |  |  |  |  |  |  |  |  |  |
| No | 1.00 |  | 1.00 |  | 1.00 |  | 1.00 |  | 1.00 |  | 1.00 |
| Yes | 2.47(1.45-4.20)^*^ |  | 1.94(1.56-2.41)^**^ |  | 3.55(2.29-5.49)^**^ |  | 1.59(1.26-1.99)^**^ |  | 2.45(1.60-3.76)^**^ |  | 1.34(1.07-1.69)^*^ |
| Hazardous drinking |  |  |  |  |  |  |  |  |  |  |  |
| No | 1.00 |  | 1.00 |  | 1.00 |  | 1.00 |  | 1.00 |  | 1.00 |
| Yes | 4.57(2.90-7.21)^**^ |  | 2.10(1.61-2.74)^**^ |  | 4.60(3.22-6.57)^**^ |  | 1.72(1.31-2.27)^**^ |  | 2.68(1.84-3.91)^**^ |  | 1.70(1.32-2.19)^**^ |
| Binge drinking |  |  |  |  |  |  |  |  |  |  |  |
| No | 1.00 |  | 1.00 |  | 1.00 |  | 1.00 |  | 1.00 |  | 1.00 |
| Yes | 3.07(1.91-4.93)^**^ |  | 2.33(1.86-2.91)^**^ |  | 5.14(3.47-7.63)^**^ |  | 2.04(1.62-2.58)^**^ |  | 3.39(2.29-5.03)^**^ |  | 1.75(1.40-2.19)^**^ |
| ART, antiretroviral treatment; HIV, human immunodeficiency virus; MSM, men who have sex with men; aOR, adjusted odds ratio; CI, confidence interval; CIE, Condomless internal ejaculation | | | | | | | | | | | |
| ^*^P < 0.05; ^**^P < 0.001 | | | | | | | | | | | |
| All models were adjusted for demographic characteristics, including age, current marital status, migrant, education attainment, employment status, and individual monthly income. | | | | | | | | | | | |

| TABLE S4 Associations of alcohol consumption patterns with ART adherence and sexual risk-taking behaviors among MSM living with HIV in Chongqing | | | | | | | | | | | |
| --- | --- | --- | --- | --- | --- | --- | --- | --- | --- | --- | --- |
| Alcohol consumption patterns | ART non-adherence |  | Having multiple sex partners |  | Engaging in commercial sex |  | Engaging in casual sex |  | Participating in group sex |  | Practicing CIE during anal sex |
|  | *aOR* (95%CI) |  | *aOR* (95%CI) |  | *aOR* (95%CI) |  | *aOR* (95%CI) |  | *aOR* (95%CI) |  | *aOR* (95%CI) |
| Alcohol use |  |  |  |  |  |  |  |  |  |  |  |
| No | 1.00 |  | 1.00 |  | 1.00 |  | 1.00 |  | 1.00 |  | 1.00 |
| Yes | 1.77(1.01-3.11)^*^ |  | 1.60(1.25-2.03)^**^ |  | 2.52(1.57-4.07)^**^ |  | 1.29(1.01-1.64)^*^ |  | 1.84(1.11-3.07)^*^ |  | 1.16(0.90-1.51) |
| Hazardous drinking |  |  |  |  |  |  |  |  |  |  |  |
| No | 1.00 |  | 1.00 |  | 1.00 |  | 1.00 |  | 1.00 |  | 1.00 |
| Yes | 3.42(2.09-5.62)^**^ |  | 1.49(1.11-2.00)^*^ |  | 3.29(2.20-4.92)^**^ |  | 1.29(0.96-1.73) |  | 2.04(1.28-3.24)^*^ |  | 1.36(1.02-1.82)^*^ |
| Binge drinking |  |  |  |  |  |  |  |  |  |  |  |
| No | 1.00 |  | 1.00 |  | 1.00 |  | 1.00 |  | 1.00 |  | 1.00 |
| Yes | 2.05(1.22-3.45)^*^ |  | 1.63(1.27-2.09)^**^ |  | 3.05(1.99-4.70)^**^ |  | 1.49(1.15-1.92)^*^ |  | 1.88(1.16-3.03)^*^ |  | 1.43(1.10-1.85)^*^ |
| ART, antiretroviral treatment; HIV, human immunodeficiency virus; MSM, men who have sex with men; aOR, adjusted odds ratio; CI, confidence interval; CIE, Condomless internal ejaculation | | | | | | | | | | | |
| ^*^P < 0.05; ^**^P < 0.001 | | | | | | | | | | | |
| All models were adjusted for age, current marital status, migrant, education attainment, employment status, individual monthly income, sexual role preference, duration since HIV diagnosis, depressive symptoms, anxiety symptoms, perceived HIV-related stigma, initiating anal sex before 18 years, recreational drug use, and HIV viral load. | | | | | | | | | | | |

| TABLE S5 Associations of AUDIT-C scores with ART adherence and sexual risk-taking behaviors among MSM living with HIV in Chongqing | | | | | | | | | | | |
| --- | --- | --- | --- | --- | --- | --- | --- | --- | --- | --- | --- |
| Alcohol consumption patterns | ART non-adherence^a^ |  | Having multiple sex partners^b^ |  | Engaging in commercial sex^c^ |  | Engaging in casual sex^d^ |  | Participating in group sex^e^ |  | Practicing CIE during anal sex^f^ |
|  | *aOR* (*95%CI*) |  | *aOR* (*95%CI*) |  | *aOR* (*95%CI*) |  | *aOR* (*95%CI*) |  | *aOR* (*95%CI*) |  | *aOR* (*95%CI*) |
| AUDIT-C score | 1.28(1.16-1.41)^**^ |  | 1.09(1.03-1.15)^*^ |  | 1.29(1.19-1.40)^**^ |  | 1.05(0.99-1.11) |  | 1.16(1.05-1.27)^*^ |  | 1.06(1.00-1.12)^*^ |
| AUDIT-C, Alcohol Use Disorders Identification Test-Consumption; ART, antiretroviral treatment; HIV, human immunodeficiency virus; MSM, men who have sex with men; a*OR*, adjusted odds ratio; *CI*, confidence interval; CIE, Condomless internal ejaculation | | | | | | | | | | | |
| ^*^*P* < 0.05; ^**^*P* < 0.001 | | | | | | | | | | | |
| ^a^adjusted for age, current marital status, migrant, education attainment, employment status, individual monthly income, sexual role preference, depressive symptoms, anxiety symptoms, perceived HIV-related stigma, initiating anal sex before 18 years, recreational drug use and HIV viral load. | | | | | | | | | | | |
| ^b^adjusted for age, current marital status, migrant, education attainment, employment status, individual monthly income, sexual role preference, depressive symptoms, anxiety symptoms, initiating anal sex before 18 years and recreational drug use. | | | | | | | | | | | |
| ^c^adjusted for age, current marital status, migrant, education attainment, employment status, individual monthly income, sexual role preference, initiating anal sex before 18 years and recreational drug use. | | | | | | | | | | | |
| ^d^adjusted for age, current marital status, migrant, education attainment, employment status, individual monthly income, depressive symptoms, anxiety symptoms, perceived HIV-related stigma, initiating anal sex before 18 years and recreational drug use. | | | | | | | | | | | |
| ^e^adjusted for age, current marital status, migrant, employment status, individual monthly income, duration since HIV diagnosis, depressive symptoms, anxiety symptoms, perceived HIV-related stigma, initiating anal sex before 18 years and recreational drug use. | | | | | | | | | | | |
| ^f^adjusted for age, current marital status, migrant, education attainment, employment status, individual monthly income, sexual role preference, duration since HIV diagnosis, depressive symptoms, anxiety symptoms, perceived HIV-related stigma, initiating anal sex before 18 years and recreational drug use. | | | | | | | | | | | |
